# Multi-Wavelength Based Optical Density Sensor for Autonomous Monitoring of Microalgae

**DOI:** 10.3390/s150922234

**Published:** 2015-09-02

**Authors:** Fei Jia, Murat Kacira, Kimberly L. Ogden

**Affiliations:** 1 Department of Agricultural and Biosystems Engineering, The University of Arizona, 1177 E. 4th Street, Shantz Building, Room 403, Tucson, AZ 85721, USA; E-Mail: feijia@email.arizona.edu; 2 Department of Chemical and Environmental Engineering, The University of Arizona, 1133 E James E. Rogers Way, Room 108, Tucson, AZ 85721, USA; E-Mail: ogden@email.arizona.edu

**Keywords:** optical density, multi-wavelength, microalgae, real-time monitoring and control

## Abstract

A multi-wavelength based optical density sensor unit was designed, developed, and evaluated to monitor microalgae growth in real time. The system consisted of five main components including: (1) laser diode modules as light sources; (2) photodiodes as detectors; (3) driver circuit; (4) flow cell; and (5) sensor housing temperature controller. The sensor unit was designed to be integrated into any microalgae culture system for both real time and non-real time optical density measurements and algae growth monitoring applications. It was shown that the sensor unit was capable of monitoring the dynamics and physiological changes of the microalgae culture in real-time. Algae biomass concentration was accurately estimated with optical density measurements at 650, 685 and 780 nm wavelengths used by the sensor unit. The sensor unit was able to monitor cell concentration as high as 1.05 g**·**L^−1^ (1.51 × 10^8^ cells·mL^−1^) during the culture growth without any sample preparation for the measurements. Since high cell concentrations do not need to be diluted using the sensor unit, the system has the potential to be used in industrial microalgae cultivation systems for real time monitoring and control applications that can lead to improved resource use efficiency.

## 1. Introduction

Microalgae have been successfully used as feedstock for the production of pharmaceutical products, nutritional supplements and chemicals [1,2,3,4]. Certain species of microalgae are candidates for the production of biofuels due to their high productivity and high oil content [5,6,7]. Producing sufficient amounts of biomass with controlled quality is the premise of production of microalgae derived products. Optimizing resource inputs and maintaining high productivity are the key components to control the quantity and cost of the algae production.

Real-time monitoring and control provides the platform to acquire the environmental and physiological dynamics of a microalgae culture system. For large scale microalgae production systems, effective decision making and overall production system management in terms of optimal resource use, harvesting and culture condition optimization (media composition, lighting, temperature, pH, dissolved oxygen levels, *etc*.) is crucial in order to achieve maximum profit and to prevent or reduce economic losses in case of contamination [8].

Measurements of biological variables, including cell mass concentration, cell size, cell morphology, population composition (*i.e.*, concerns with contamination), pigments and lipid content, are especially desirable because they are the direct indicators of the dynamics of a microalgae culture system. Standard methods developed for measurements of these variables are either too laborious or destructive to be employed for real-time monitoring and control purposes [9,10]. Spectrophotometry has been widely used to estimate these biological variables by measuring the absorbance, turbidity or fluorescence of the culture suspension [11,12,13]. As a non-destructive and rapid analytical method, spectrophotometry became a preferable candidate for real-time monitoring and control of microalgae culture systems.

There are some commercialized sensors to monitor microalgae concentration [14,15,16,17]. However, most of them are designed to monitor microalgae concentration at an environmental level which is much lower than the cell concentration in microalgae production applications. Furthermore, these sensors are too expensive for low added value product applications. Therefore, they are not practical to integrate into outdoor raceway or photobioreactor based algae production systems. 

There have been only few studies on development and evaluation of self-constructed optical sensors for microalgae monitoring and control applications [18,19,20,21,22,23,24,25]. For instance, Sandes *et al*. [23] focused on measuring the intensity of light transmitted through a transparent production tube with a 10 mm light path length containing a microalgae suspension using a LED (880 nm) and photodiode pair mounted on the opposite side of the tube. The sensor was able to estimate the cell concentration of *Nannochloropsis oceanica* and correlated both with dry weight (up to 2.0 g·L^−1^) and cell count. Briassoulis *et al*. [18] developed an automated flow-through density sensor and harvesting system for *Nannochloropsis* sp. The LEDs paired with photosensors were used to measure the light transmittance of cell culture at 470, 518, 630 and 940 nm. A neural network was employed to estimate biomass concentration by associating the voltage readings from each photosensor with the cell concentration measured by cell count. The sensor reported has an absolute estimation error below 8 × 10^6^ cells·mL^−1^, and a maximum error at 9% within interval of 5 to 145 × 10^6^ cells·mL^−1^. Nedbal *et al*. [22] described the monitoring of chlorophyll concentration and cell density of a cyanobacterial suspension by a flat-cuvette photobioreactor with a built-in fluorometer and densitometer. Blue LEDs (455 nm) and orange LEDs (627 nm) were used for excitation of blue absorption and phycobilins, respectively. The optical density of the suspension was measured at 680 nm and 735 nm. Cell count and chlorophyll concentration were linearly proportional to optical density (OD) 680 in the range 0.1–1.2 and to OD 735 in the range 0.02–0.4; these values of OD or cell density are typically exceeded in microalgae production systems. Furthermore, the sensor unit was designed for a specific PBR, re-configuration and re-calibration of the sensor will be necessary if it were to be integrated into other culture systems. Marxen *et al*. [20] developed a bioreactor system for the cultivation of *Synechocystis* sp. PCC6803. Dry mass of microalgae was estimated by the measurement of optical density of the suspension at 870 nm. Chlorophyll concentration was determined by the pulse amplitude modulation (PAM) technique. Yao *et al*. [25] developed and tested an optical density based sensor using a LED and photodiode based unit at two wavelengths (Red and NIR) to monitor algae growth. The sensor was able to estimate biomass concentration ranging from 0.05 to 0.50 OD in indoor conditions. The study reported temperature dependency of the sensor unit that caused inaccurate measurement of algal biomass concentration when tested in outdoor conditions. 

To our knowledge, there is no current optical sensor design that exists in the market for measuring multiple biological parameters in real time both in an indoor PBR and outdoor raceway system within a high cell concentration range and without needing sample preparation (*i.e.*, dilution, washing, filtration) prior to measurements. Therefore, we describe here the design, development and evaluation of a relatively low cost multi-wavelength laser diode-photodiode based sensor applicable for use both in an indoor photobioreactor system and an outdoor raceway system to monitor optical density and growth of microalgae in real time. 

## 2. Material and Methods 

### 2.1. Optical Density Measurement Sensor 

**Figure 1:**
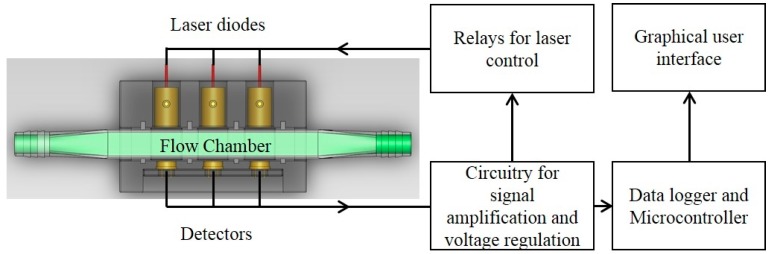
Component layout of the optical sensor unit. Three laser diodes at wavelengths of 650 nm, 685 nm and 780 nm were aligned with 3 photodiodes with a detection range of 350–1100 nm. The flow chamber window was perpendicular to the laser beam.

The growth dynamics of the microalgae culture was measured using the real-time optical density sensor (Figure 1) developed in this study. Light absorbance of microalgae suspensions at multiple wavelengths correlate to different characters of microalgae cells. The 650 (650 nm–10 mW, US-Lasers Inc., Baldwin Park, CA, USA), 685 (HL6750MG, Oclaro Inc., San Jose, CA, USA) and 780 (780 nm–10 mW, US-Lasers Inc., Baldwin Park, CA, USA) laser diodes were used in the developed sensor unit for this study. These three wavelengths have been commonly used to estimate the cell concentration of microalgae suspension [11,12,13]. Light absorbance at 780 nm estimates the turbidity of the suspension since the color of microalgae has no effect on the absorbance, whereas, light absorbance at 650 and 685 nm correlates to both intensity of the color (*i.e.*, chlorophyll content) and cell concentration. 

The optical sensor unit consisted of laser diode modules as light sources, a photodiode as a detector and custom-made fixtures to house them. Laser diode modules consisted of laser diodes, driver circuit (iC-WK BMST WK2D, iC Haus LLC., Jaffrey, NH, USA) and brass housing with adjustable optical lenses (10.4 mm Module Housing Kit, US-Lasers Inc., Baldwin Park, CA, USA). An optical filter (86734, Edmund Optics Inc., Barrington, NJ, USA) was placed in front of the 685 nm laser diode to allow only the light with wavelength from 680 to 690 nm to pass through. The system design enabled adjustment of the output power of the modules by a potentiometer connected to a 5 V DC power source. The photodiodes (FDS100, Thorlabs Inc., Newton, NJ, USA) with a detection range of 350–1100 nm were connected to a zero-bias amplification circuit. Three pairs of laser diode modules and photodiodes were placed in a linear pattern in the fixture. Each pair was aligned and placed 15 mm apart. The diameter of the circular light beam from the laser diode modules was adjusted to be slightly smaller than the size of detection window on the photodiode. The optical sensor unit was designed to enable measurements from either standard cuvettes or custom made flow chambers with a light path length of 5 mm. Cuvettes and flow chambers were placed perpendicular to the laser beam and 1 mm away from the window of photodiodes. When used for real-time monitoring, laser diodes were powered sequentially by the data logger’s control module to avoid light noise from individual laser light sources. The voltage generated from the photodiodes was amplified and recorded by a data logger and controller (CR3000, Campbell Scientific Inc., UT, Logan, UT, USA). The entire sensor unit was mounted in a weather proof enclosure enabling connection of tubes for algae flow through the sensor flow cell and signal cables for the laser diodes and photo diodes.

The voltage output of the photodiode is proportional to the intensity of incident light. According to Beer-Lambert law, the light absorbance of the sample was determined by,

$$Abs=-\ln\left(V_{s}/V_{b}\right)$$

*Abs* = Light absorbance;
*V_b_* = Output of the photodiode from growth media (mV);
*V_s_* = Output of the photodiode from a sample (mV).

### 2.2. Cultivation Conditions and Organisms

#### 2.2.1. Indoor Photobioreactor (PBR) Cultivation

*Chlorella sorokiniana* (DOE 1412) received from Pacific Northwest National Laboratory, Richland, WA, USA [26] was cultivated in local well water enriched with Peters professional® 20-20-20 general purpose water soluble fertilizer 0.26 g·L^−1^ , Citraplex 20% iron 0.053 g·L^−1^ (Citraplex 20% Iron, Loveland Products Inc., Loveland, CO, USA) and trace elements solution (H_3_BO_3_ 0.0029 g·L^−1^, MnCl_2_•4H_2_O 0.0018 g·L^−1^, ZnSO_4_•H_2_O 0.00014 g·L^−1^, Na_2_MoO_4_•2H_2_O 0.00039 g·L^−1^, CoCl_2_•6H_2_O 0.000055 g·L^−1^) under illumination intensity of 200 µmol·m^−2^·s^−1^ or 400 µmol·m^−2^·s^−1^ in rectangular air lift photo bioreactors (PBRs). The algae culture temperature was light intensity dependent and was stabilized at 30 ± 2 °C. The pH of the medium was controlled at 7 ± 0.3 by injecting CO_2_ from a pressurized liquid CO_2_ tank into PBRs. 

#### 2.2.2. Outdoor Open Pond Raceway Cultivation

*Scenedesmus obliquus* was used in the outdoor open pond raceway cultivation experiments. *Scenedesmus obliquus* was received from Texas A&M AgriLife Research (Pecos, Texas, USA) and was cultivated in local well water enriched with Pecos medium, trace metal solution and 5g·L^−1^ NaCl. The Pecos medium contained 0.1 g·L^−1^ urea ((NH_2_)_2_CO), 0.012 g·L^−1^ MgSO_4_•7H_2_O, 0.035 g·L^−1^ NH_4_H_2_PO_4_, 0.175 g·L^−1^ Potash (KCl), 0.0054 g·L^−1^ FeCl_3_ and 0.02 g·L^−1^ Na_2_CO_3_. The culture was maintained in an open pond paddle wheel raceway with a surface area of 3 m^2^ located in Tucson, Arizona, USA. The culture depth was maintained at 10 cm and increased to 15 cm later in the experiment. The pH of the medium was controlled at 8 ± 0.05 by injecting 95% CO_2_ through an air sparger. 

### 2.3. Offline Biomass Concentration Measurement

Biomass concentration of microalgae was determined by both cell counting and ash-free dry weight (AFDW) measurements. Cell suspension was diluted to a concentration between 10^6^ and 10^7^ cells·mL^−1^ for cell counting by a neubauer chamber hemocytometer (Hy-Lite Ultra-plane, Clayadams, Franklin Lakes, NJ, USA) under a microscope (XSZ-138, AOK International Group Ltd., Shanghai, China). The AFDW of the cells was measured following the method described by Zhu and Lee (1997) [27]. The light absorbance of the cells suspension was measured at 650, 685, 750 and 780 nm by a spectrophotometer (DR 3800, HACH, Loveland, CO, USA) using a 10 mm light path length cuvette. Samples were diluted with deionized water when necessary to keep the absorbance reading below 0.5. 

### 2.4. Real-Time Monitoring of Microalgae Growth Dynamics

#### 2.4.1. Indoor PBR Cultivation

The microalgae culture system consisted of an air lift flat panel PBR illuminated by a fluorescent lighting system. The pH (HI1001, Hanna Instruments, Woonsocket, RI, USA), electrical conductivity (HI3001, Hanna Instruments, Woonsocket, RI, USA), dissolved oxygen (DO1200/T, Sensorex, Garden Grove, CA, USA) and thermocouple temperature probes (Type T, Omega Engineering Inc., Stamford, CT, USA) were placed in the PBR for monitoring and control by a CR3000 datalogger. Each sensor was scanned every second and 10 minute averaged data was stored in the datalogger.

**Figure 2:**
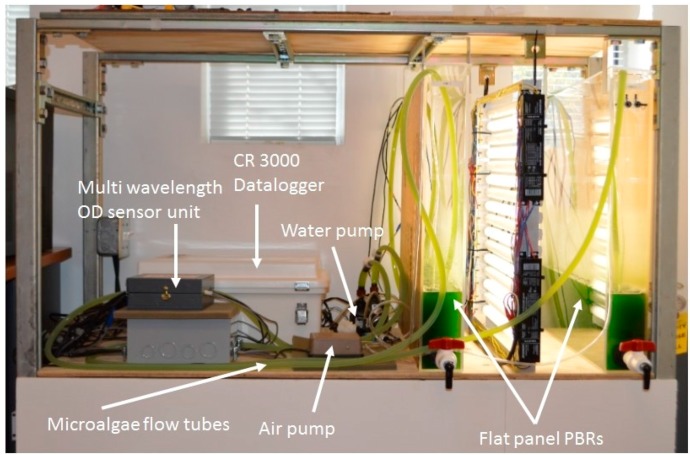
Multi wavelength optical sensor integrated into air-lift flat panel photobioreactors for real-time microalgae growth monitoring.

Flat panel PBRs with dimensions of 61 (H) × 61 (L) × 7.6 cm (W) were built using 6.35 mm thick clear acrylic panels (ACRYCLR0.250PM48X48, Plexiglas, San Diego, CA, USA). Air was constantly injected into the PBR via a 45.7 cm long air sparger mounted at the bottom of PBR for aeration and to achieve proper mixing of the microalgae culture. Carbon dioxide injection was controlled by the datalogger to maintain a desired pH level (7 ± 0.3) in the PBR. The lighting system consisted of sixteen 61 cm 17-watt fluorescent light tubes (F17T8/741, Litetronics International, Inc., Harvey, IL, USA) mounted on a supporting structure. Two levels of light intensity (200 and 400 µmols·m^−2^·s^−1^) were achieved by adjusting the number of lights used. The light remained on 24 hours per day, no dark period was used. A small centrifugal pump (Seltz 20, Hydor, Sacramento, CA, USA) was used to recirculate cell suspension through the inline optical density measurement unit for the PBR. The optical density sensor was connected to the PBR system for continuous monitoring of OD and microalgae growth (Figure 2).

#### 2.4.2. Outdoor Open Pond Raceway Cultivation

**Figure 3:**
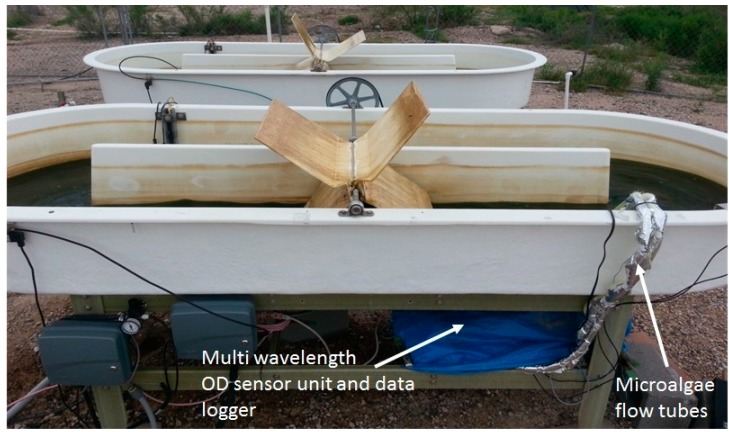
Optical sensor integrated into an open pond raceway for real-time microalgae growth monitoring.

The optical density sensor was also integrated into an outdoor raceway system for continuous monitoring of microalgae growth (Figure 3). Since sensor electronics maybe sensitive to environmental conditions, the optical sensor with its housing and the datalogger were placed in a location at the outdoor raceway site to minimize direct exposure to sunlight. The laser output is also temperature dependent (5–15 mV/°C, vary with lasers). Therefore a temperature control unit was installed and consisted of a small heater plate (HT24S, Thorlabs, Newton, NJ, USA) and heat sink (55 mm Fan Heatsink, Amazon.com, USA) to maintain a constant temperature (25 ± 0.1 °C) inside the sensor box. This also ensured a constant laser power output. The paddle wheel in the raceway system was operated 24 hours a day for continuous culture mixing. The CO_2_ injection was turned off during night time. In addition to the measurement data collected for the indoor experiment, photosynthetically active radiation (PAR) was also measured using a quantum sensor (SQ-110, Apogee instruments, Logan, UT, USA) at the level of the raceway system. All variables were recorded at the same frequency for sensor scanning and data averaging as described for the indoor cultivation experiment. The experiment occurred from 2/25 to 3/15 for a total of 18 days.

## 3. Results and Discussion

### 3.1. In Situ Calibration of the Optical Density Measurement Unit

Light absorbance from a flowing cell suspension can be different from static samples due to cell movement and potentially the presence of fine air bubbles. Therefore, a calibration of the unit using flowing microalgae culture is necessary. In order to achieve in-line real-time monitoring, sample preparation needs to be eliminated or automated. In this study, flow chambers with light path lengths of 5 mm were used to extend the measurement range of the unit without requiring sample dilution.

**Figure 4:**
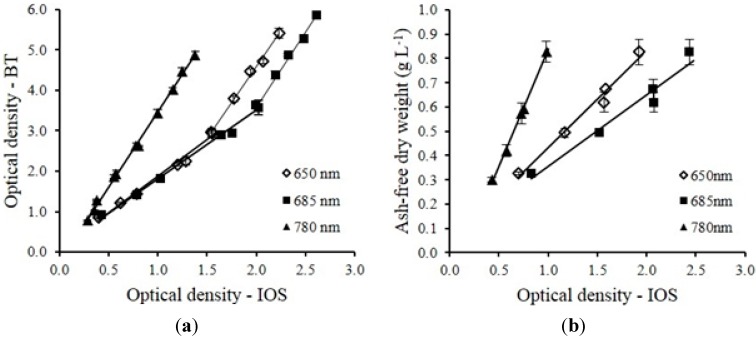
(**a**) Correlation between the optical densities of DOE 1412 in the PBR measured by a bench-top spectrophotometer (BT) and by the inline optical sensors (IOS). OD_650 (BT)_ = 1.82 × OD_650 (IOS)_ + 0.056 (AFDW < 0.592 g·L^−1^), OD_685 (BT)_ = 1.70 × OD_685 (IOS)_ + 0.11 (AFDW < 0.592 g·L^−1^), OD_650 (BT)_ = 3.54 × OD_650 (IOS)_ − 2.51 (0.592 g·L^−1^ < AFDW < 1.05 g·L^−1^), OD_685 (BT)_ = 3.72 × OD_685 (IOS)_ − 3.88 (0.592 g·L^−1^ < AFDW < 1.05 g·L^−1^), OD_780 (BT)_ = 3.71 × OD_780 (IOS)_ − 0.2445 (AFDW < 1.05 g·L^−1^). (**b**) Correlation between optical density (IOS) and AFDW, AFDW = 0.96 × OD_780 (IOS)_ − 0.12 (R^2^ = 0.99); AFDW = 0.40 × OD_650 (IOS)_ + 0.032 (R^2^ = 0.98); AFDW = 0.30 × OD_685 (IOS)_ + 0.061 (R^2^ = 0.96).

The optical sensor unit (Figure 1) developed in this study (referred as IOS hereafter) was calibrated by comparing the reading from the sensor unit to that from a bench-top spectrophotometer (referred as BT hereafter) at 650, 685 and 780 nm. The bench-top spectrophotometer (DR3800, Hach, CO, USA) was calibrated to both ash-free dry weight (AFDW) and cell count (CC) for *C. sorokiniana* at all three wavelengths: AFDW = 0.188 × OD_650_ + 0.0453 g·L^−1^ (R^2^ = 0.96), AFDW = 0.161 × OD_685_ + 0.0292 g·L^−1^ (R^2^ = 0.96), AFDW = 0.205 × OD_780_ + 0.0546 g·L^−1^ (R^2^ = 0.95), CC = (28.6 × OD_650_ + 1.13) × 10^6^ cells·mL^−1^ (R^2^ = 0.91), CC = (26.8 × OD_685_ − 3.92) × 10^6^ cells·mL^−1^ (R^2^ = 0.95), CC = (29.8 × OD_780_ + 3.96) × 10^6^ cells·mL^−1^ (R^2^ = 0.90). The optical density readings measured from the spectrophotometer using standard 10 mm cuvettes were compared to the results obtained from optical sensor unit using 5 mm flow cell. Strong linear correlations between the two measurement units were obtained at all wavelengths examined (Figure 4). A linear correlation was tightly followed (R^2^ = 0.99) between the optical density measurements obtained from IOS and BT units at 780 nm with cell concentration up to 1.05 g·L^−1^ (1.51 × 10^8^ cells·mL^−1^). Linear correlations hold for OD_650_ (R^2^ = 0.98) and OD_685_ (R^2^ = 0.99) for cell concentrations below 0.592 g·L^−1^. However, beyond this range while below 1.05 g·L^−1^, different linear correlations were observed for these two wavelengths (Figure 4). Compared to the results from Nedbal *et al*. [22], the optical sensor unit showed the capability of measuring cell concentration over a wide range without dilution of the samples. The same calibration procedure was performed for *S. obliquus* during outdoor testing.

**Figure 5:**
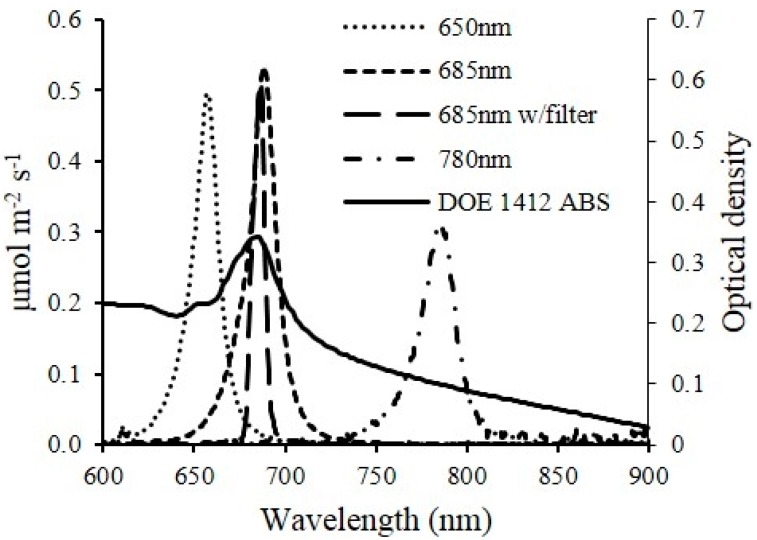
Light absorbance spectrum of DOE 1412 and light spectra of laser diodes used on the optical sensor.

The OD readings from the optical sensor unit measured using 5 mm path length flow cell should be half of that from the spectrophotometer using a standard 10 mm cuvette in theory. However, the results did not show an exact correlation between the two units. This was because of the light quality from the laser diodes wasn’t the same as that in a spectrophotometer where a monochromatic light was generated. Figure 5 shows the spectra of the laser diodes used in the developed sensor unit, measured by a spectroradiometer (PS-300, Apogee Instruments, Logan, UT, USA) and the optical density spectra of DOE 1412. The peak wavelengths of each laser diode were slightly shifted from what was claimed by the manufacturers. An optical filter (86734, Edmund Optics, Barrington, NJ, USA) was used to narrow the band width of 685 nm laser diode from 80 nm to 10 nm and corrected the peak wavelength back to 685 nm from 688 nm. Despite the inferiority of the light beam generated from laser diodes, the strong linear correlations proved that the optical sensor unit was able to estimate the cell density as accurate as a spectrophotometer via calibration (Figure 4). 

### 3.2. Real-Time Microalgae Growth Monitoring

**Figure 6:**
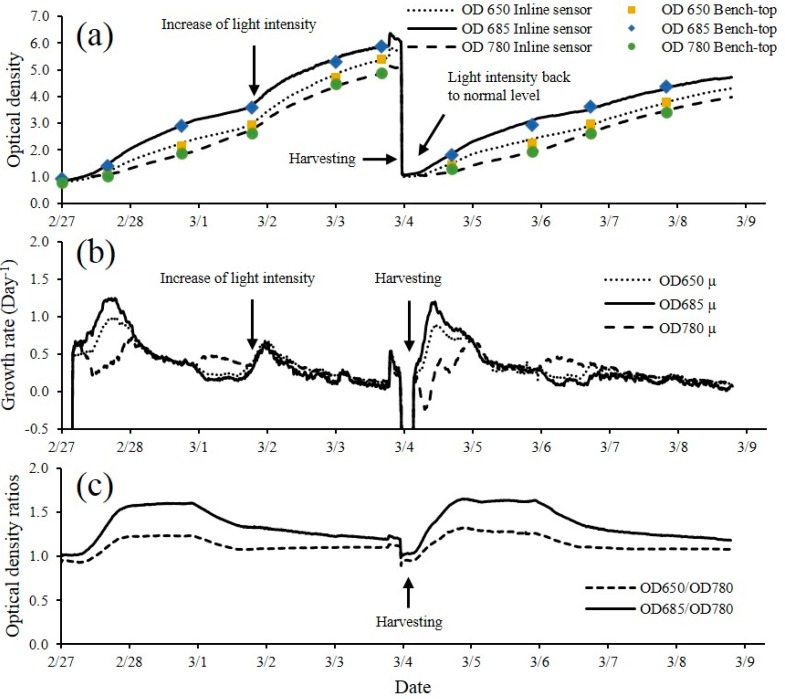
(**a**) Dynamics of optical density at 650 nm, 685 nm and 780 nm during semi-continuous culture of DOE 1412 run for 10 days. Illumination intensity was increased from 200 µmol·m^−2^·s^−1^ to 400 µmol·m^−2^·s^−1^ during the first batch on 3/2, it was then reduced to 200 µmol·m^−2^·s^−1^ by the end of the batch; (**b**) Growth rate of DOE 1412 at 650, 685 and 780 nm and (**c**) ratios of optical densities at 650/780 nm and 685/780 nm.

The optical sensor unit along with other sensors to monitor algae culture environment was integrated into a PBR to monitor the dynamics of a microalgae culture system. Figure 6a shows the growth dynamics of semi-continuous culture of DOE 1412 as measured by the optical sensor unit over a period of 10 days. Sensor output shown in Figure 6a was calibrated to optical density reading from a bench-top spectrophotometer. The optical sensor unit showed the capability to capture the growth phases during semi-continuous operation, and the sudden change of cell concentration due to harvesting and addition of fresh media (indicated with arrows on the Figure). A maximum cell concentration of 1.05 g·L^−1^ (1.51 × 10^8^ cells·mL^−1^) was observed during the cultivation experiment without any sample preparation and dilution for the measurements. 

Growth dynamics of the microalgae was quantified by the growth rate. The growth rate was determined by the following equation with Δt of 2 hours (0.08 days).

$$\mu =\frac{\ln{\left(OD_{2}\right)}_{\lambda }-\ln{\left(OD_{1}\right)}_{\lambda }}{\text{Δ}t}$$

µ = Growth rate (day^−1^);
OD = Optical density of microalgae at different time points (λ = 780 nm);
Δt = Difference between the two time points (day).

The change of growth rate was clearly demonstrated by plotting the growth rate (µ) of DOE 1412 over time (Figure 6b). The initial lag phase was followed by an increase in cell growth. Microalgae culture reached maximum growth rate soon after the lag phase when there is no light limitation. The growth rate then gradually decreases as the culture becomes light limited. When the illumination intensity was increased from 200 µmol·m^−2^·s^−1^ to 400 µmol·m^−2^·s^−1^ on 3 February 2014 an increase in growth rate was observed (Figure 6b). The growth rate dropped down to the level prior the alternation of light intensity as the culture again became light limited. These events were detected by the optical sensor unit (Figure 6a,b). Although real time growth rate is not required for microalgal biomass production purposes, data with such high resolution provided a great tool for studying the fast response of microalgae to sudden change of the environmental conditions. 

Monitoring not only the cell concentration change, but also the dynamic physiological status of the microalgae culture including the changes in growth rate and the change of chlorophyll content can serve as indicators of the health of the culture. This is important for cultivation of microalgae production when it is desirable to control conditions to produce a product of interest. For example, some microalgae produce more lipids when nutrients such as nitrogen are limiting. The ratios of optical densities at different wavelengths (685/780 nm and 650/780 nm) are shown in Figure 6c. The ratios remained constant during lag phase, followed by a rapid increase during the exponential growth phase and stabilized at a higher level throughout the linear growth phase. The ratios then started to decrease as the growth of cells slowed down which indicated the transition from linear to stationary phase. The pattern of the ratio change occurred repeatedly over the time course of the experiment regardless of the growth pattern change induced by increased light intensity during the first batch. Signaling of this transition indicated that there is a decrease of chlorophyll content which absorbs most of the red light during the period indicated by the decreasing optical density ratios [28]. This might have been due to nitrogen limitation, since nitrogen is often rapidly consumed by algal cells during exponential growth according to López *et al*. [19]. Similar results for the change of OD 680/ OD 735 was reported by Nedbal *et al*. [22]. 

**Figure 7:**
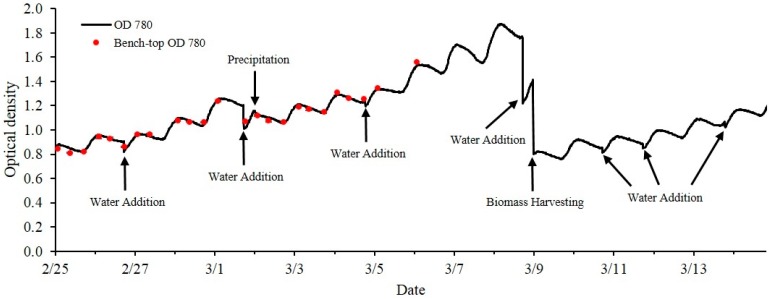
Optical density change of *S. obliquus* in open pond raceway over 18 days. Black arrows indicate events of water addition, precipitation and biomass harvesting.

The optical sensor unit was also integrated into an outdoor open pond raceway for stability testing under highly dynamic outdoor weather conditions such as large temperature variations between daytime and nighttime periods. For instance, a 20 °C temperature difference were measured inside sensor box from daytime to nighttime when the temperature control system was not activated. The optical density of the culture of *S. obliquus* during a period of 18 days recorded by the optical sensor is shown in Figure 7. The real-time optical density shows repeatedly an increase OD reading indicating the biomass increase during the day time due to photosynthesis. A small decrease in optical density was observed during the nighttime since photosynthetic microorganisms metabolize intracellular carbohydrate to sustain their metabolic activity as described by Ogbonna and Tanaka [29]. Sudden decreases of optical density of the culture due to water addition, precipitation (rain) and biomass harvesting were clearly shown in the Figure 7 labeled by arrows. 

**Figure 8:**
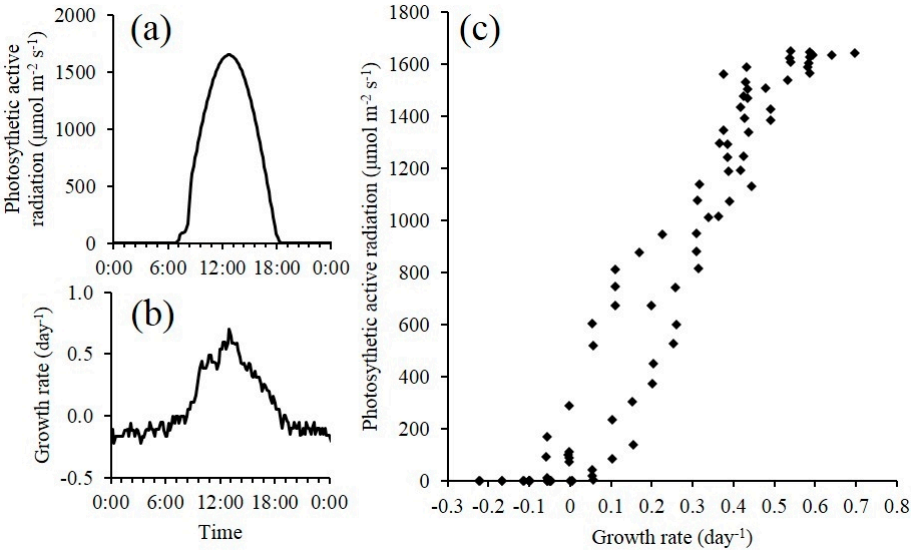
(**a**) Photosynthetic active radiation (PAR) of a sunny day in Tucson, AZ, USA; (**b**) Growth rate (µ) of *S. obliquus* in open pond raceway of the same day; (**c**) Scattered plot of PAR and µ from the data presented in (**a**,**b**).

The growth rate of *S. obliquus* was compared to photosynthetic active radiation (PAR) measured at the raceway (Figure 8). The growth rate of *S. obliquus* was dependent on the PAR level except during the water addition time period. This set of high resolution data enables one to evaluate in detail about how *S. obliquus* responds to solar radiation level in a sunny day. The correlation between PAR and growth rate can be used for the prediction of biomass production rate based on historical weather data for a given region. 

## 4. Conclusions

The multi-wavelength laser diode based optical sensor unit was designed, developed and evaluated for the monitoring of microalgae culture dynamics in real-time both in a PBR and in an outdoor raceway system. The optical sensor unit prototype demonstrated the capability of estimating cell concentration and changes of the physiological status of the microalgae culture in real-time. The sensor unit was operated continuously for 18 days without any visible microalgae biofilm deposit observed on the flow chamber of the sensor unit. In this design, the only sensor hardware part that had contact with culture medium was the flow chamber which can be easily replaced. For industrial microalgae production, the application of ultra-hydrophobic material (Hydrophobic glass coating, Ultra Tech International, Inc., Jacksonville, FL, USA) on the surface of flow chamber can further extend the maintenance interval. Biomass concentration was accurately estimated by optical density measurement at 650, 685 and 780 nm wavelengths. The sensor was capable of measuring maximum optical density of 5.41, 5.86 and 4.88 without sample preparation at 650 nm, 685 nm and 780 nm respectively. Growth rates and ratios calculated from optical density at each wavelength were good indications for monitoring of microalgae growth transitions and for detection of disturbances to the culture system (*i.e.*, change of light intensity, water addition, rain, and harvesting). A temperature control device for the sensor is necessary, especially for outdoor applications where air temperature can vary significantly, since the output power of laser diodes were temperature dependent. The cell concentration measurement range can be further improved by shortening the light path length of the flow chamber. Other laser modules and wavelengths of interest can be added to expand the number of biological variables measured by the sensor which is our focus for future studies. The real-time monitoring data from the optical sensor can be valuable for microalgae modeling studies both for PBR and outdoor raceway based production systems. With proper calibration, installation and operation, the optical sensor described in this study can be integrated into microalgae culture systems for monitoring and control purposes at a relative low cost to ultimately help optimize product quality and quantity. 
